# Integrating the “Quit and Stay Quit Monday” Model into Smoking Cessation Services for Smokers with Mental Health Conditions: A Pilot Randomized Controlled Trial

**DOI:** 10.1155/2023/8165232

**Published:** 2023-07-22

**Authors:** Mahathi Vojjala, Christina N. Wysota, Ololade Oketunbi, Quiann King, Erin S. Rogers

**Affiliations:** ^1^NYU Grossman School of Medicine, Department of Population Health, New York, NY, USA; ^2^NYU School of Global Public Health, New York, NY, USA; ^3^Department of Prevention and Community Health, Milken Institute School of Public Health, George Washington Cancer Center, George Washington University, Washington, DC, USA; ^4^NYU Silver School of Social Work, Substance Abuse Research Education & Training Program, USA; ^5^NYU College of Arts and Sciences, New York, NY, USA

## Abstract

**Introduction:**

People with mental health conditions (MHCs) are less likely to achieve long-term abstinence than people without MHCs. The Quit and Stay Quit Monday (*QSQM*) model offers a long-term approach to treating tobacco use by encouraging people to quit, requit, or recommit to quit smoking every Monday.

**Aim:**

To evaluate the efficacy, patient satisfaction, and patient engagement with an intervention that integrated the *QSQM* model into multicomponent smoking cessation services among people with an MHC.

**Methods:**

This was a randomized controlled pilot trial. Eligibility criteria were as follows: (1) ≥18 years old, (2) smoked a cigarette in the past 30 days, (3) diagnosis of an ICD-10 MHC, (4) interest in quitting smoking, (5) able to receive services in English, and (5) had an active email and a cell phone. The intervention group (*n* = 33) received *QSQM*-focused telephone coaching, a weekly *QSQM* email newsletter, a SmokefreeTXT anchored around a Monday quit date, and 4 weeks of nicotine replacement therapy (NRT). The control group (*n* = 36) received information about contacting their state Quitline for usual services. Primary outcomes were self-reported quit attempts, 7-day abstinence, and intervention satisfaction at 3 months.

**Results:**

Twenty-four participants (73%) in the intervention group began telephone coaching, 26 (79%) enrolled in the *QSQM* email newsletter, 19 (58%) enrolled in SmokefreeTXT, and 15 (46%) used NRT. Using a penalized intent-to-treat approach, quit attempts in the intervention and control groups were 63.6% and 38.9% (OR 2.75, 95% CI 1.03-7.30), respectively. Seven-day abstinence in the two groups was 12.1% and 5.6% (OR 2.35, 95% CI 0.40-13.74), respectively. Of the 15 intervention group participants who set a quit date during the intervention, 13 (86.7%) selected a Monday quit day. Qualitative interviews revealed positive participant experiences with picking a Monday quit day. On follow-up surveys, 89.5%, 69.3%, and 64.3% of intervention participants reported that the counseling, *QSQM* email, and text messaging, respectively, were very or somewhat helpful.

**Conclusions:**

The *QSQM* model was acceptable and potentially efficacious among people with MHCs, but intervention engagement and satisfaction were modest. Future research should adapt or develop new *QSQM* delivery approaches to improve patient engagement and potential efficacy of the model. This trial is registered with clinicaltrials.gov (NCT04512248).

## 1. Introduction

People diagnosed with a mental health condition (MHC) are more likely to smoke cigarettes than people without a mental health diagnosis [1]. Randomized controlled trials (RCTs) support the effectiveness of smoking cessation counseling combined with nicotine replacement therapy (NRT) for people with MHCs who smoke [2–5]. However, most people with MHCs have difficulty sustaining long-term abstinence, even after multiple quit attempts [1, 6, 7]. Novel longitudinal treatment approaches are needed to help people with MHCs quit smoking.

The Quit and Stay Quit Monday (*QSQM*) model is a longitudinal smoking cessation approach designed by *The Monday Campaigns* that encourages people to quit, requit, or recommit to quit every Monday [8]. The model leverages people's natural tendencies and preferences to pursue smoking cessation information on Mondays more than any other day [9]. The model combines a weekly cue of quitting on Monday and the introduction of tobacco cessation messaging that encourages consistent healthy behavior each Monday [8]. The model promotes each Monday as a “fresh start,” thereby providing 52 cues to quit each year to support a more sustainable commitment to quitting compared to models that rely on time-limited episodes of care [8, 10]. Researchers at Johns Hopkins University conducted a pilot study of the *QSQM* model and found that participants in groups that encouraged using Monday as a quit day were more likely to select a Monday as their quit day and to report higher confidence in quitting as compared to participants in a control group [10].

The *QSQM* model is a promising approach for helping people with MHCs quit. The model's longitudinal orientation treats tobacco use as a chronic relapsing condition, which may be helpful for people with MHCs who require longer-term support to sustain abstinence [11]. The longitudinal approach can normalize relapse and limit the abstinence violation effect [12, 13] by guiding participants who relapse or slip to view every Monday as a cue to start again on the path to abstinence. Lastly, the model guides longitudinal behavior change without requiring significant resources or cognitive effort, giving it strong potential for wide-scale dissemination when integrated into existing cessation interventions, such as text-messaging and telephone coaching programs.

There are currently no studies evaluating the *QSQM* model with people who have an MHC. The purpose of this study was to evaluate the preliminary efficacy, patient satisfaction, and patient engagement with an intervention that integrated the *QSQM* model into multicomponent smoking cessation services among people with an MHC. The NYU Langone Health IRB approved the study (#s20-01247).

## 2. Methods

### 2.1. Study Design, Setting, and Participants

The study used a two-group, parallel-randomized RCT design. People were eligible if they (1) were age ≥ 18 years old, (2) had smoked a cigarette in the past 30 days, (3) had received an ICD-10 mental health diagnosis or received care from a psychiatrist within the NYU Langone Health (NYULH) system in the prior 12 months, (4) were interested in quitting smoking, (5) were comfortable receiving services in English, (6) had a cell phone that could receive text messages, and (7) had an email address. People who reported pregnancy or breastfeeding were excluded.

To recruit participants, study staff used NYULH's DataCore services to generate a list of patients from NYULH's electronic health record system, Epic (Epic Systems Corporation), who had received an ICD-10 mental health diagnosis or were seen in a mental health clinic and were screened as current tobacco users during a clinical visit in the last 12 months. Staff mailed potential participants a letter and study flyer in the mail and subsequently called each patient two weeks later to discuss the study and screen for eligibility. After confirming eligibility, the study staff obtained verbal informed consent from all participants following an IRB-approved consent script. Using computer-generated random numbers, a research assistant administered a baseline survey and randomized participants 1 : 1 to intervention or control.

### 2.2. Interventions

#### 2.2.1. Quit and Stay Quit Monday Intervention Group

The intervention included four components:

1. *Telephone coaching*: participants were offered four telephone-based smoking cessation coaching sessions over 6-8 weeks following a protocol adapted from prior studies [2, 5, 14]. The protocol followed clinical practice guidelines for the treatment of tobacco use [5] and included problem-solving therapy and motivational interviewing approaches to help participants select a quit date and develop an individualized quit plan. The protocol was adapted for the current trial to incorporate the *QSQM* approach. The first telephone coaching session was scheduled on a Monday. The telephone coach encouraged participants to select a future Monday as their quit date and to use every Monday as a day to quit, requit, or recommit to quitting, even after coaching discharge. Because each individual's quit process is different, the timing of each session varied, but the coach aimed to not let more than two weeks pass in between sessions. At the conclusion of a completed session, the subsequent session was scheduled based on the participant's progress
2. *Quit and Stay Quit Monday email newsletter*: the intervention coach offered to enroll participants in a *QSQM* email newsletter developed and managed by *The Monday Campaigns* [8]. The newsletter sends weekly quitting tips and support each Monday. Participants could unsubscribe from the emails at any time
3. *Monday-anchored SmokefreeTXT*: the intervention coach offered to enroll participants into the National Cancer Institute's (NCI's) SmokefreeTXT program to receive supportive smoking cessation text messages. SmokefreeTXT provides two weeks of prequit messages before a participant's quit date and six weeks of messages after a participant's quit date. For the current study, the intervention coach entered each participant's selected Monday as their quit day during SmokefreeTXT enrollment, so that the program's supportive texts would anchor around a Monday quit day. Participants could opt-out of receiving the text messages at any time
4. *Nicotine replacement therapy*: the intervention offered a free 4-week supply of NRT to participants who did not have medical contraindications [5]. Participants smoking less than 10 cigarettes per day received a single NRT: patch (14 mg), gum (4 mg or 2 mg depending on time to first cigarette), or lozenge (4 mg or 2 mg depending on time to first cigarette). Participants smoking 10 or more cigarettes per day received a combination NRT: patch (21 mg) plus gum or lozenge (2 mg or 4 mg depending on time to first cigarette).

#### 2.2.2. Control Group

Participants randomized to the control arm were given information about how to contact the New York state (NYS) Quitline to receive telephone coaching, NRT, and other cessation services.

#### 2.2.3. Assessments and Measures

*(1) Participant Characteristics and Tobacco Use*. Participants completed a telephone survey after enrollment (before randomization) assessing sociodemographics, current and historical smoking [15], and nicotine dependence [16]. Participants' mental health diagnoses (ICD-10 codes) in the 12 months prior to enrollment were obtained from NYULH Epic data pulls conducted by NYULH's DataCore service. Participants in both groups completed telephone surveys at three months with a blinded research assistant to assess past 7-day cigarette use and quit attempts greater than 24 hours.

*(2) Intervention Satisfaction and Engagement*. The literature recommends using mixed-method approaches to assess objective and subjective intervention engagement, including the following: (1) the *extent of intervention usage* (e.g., amount, frequency, and duration) and (2) *subjective intervention experience* (e.g., satisfaction, attention, and interest) [17–19]. Following these recommendations, we used intervention process data as an objective measure of the extent of intervention usage by participants. Study staff documented each participant encounter using standardized electronic notes. The notes captured the date and length of each coaching session, topics covered, and intervention process measures including whether the participant was provided with NRT, whether the participant selected a quit date, and whether the participant was enrolled in the *QSQM* emails and/or SmokefreeTXT. To assess participants' subjective experience with each intervention component, the 3-month follow-up survey gathered information about use and satisfaction with each intervention component, selection of a Monday quit date, and open-ended questions gathering their feedback about the intervention components (what was helpful, what was not helpful, and how did they feel about being asked to select a Monday quit date). Participants received a $15 gift card for each survey completed.

#### 2.2.4. Outcomes

The study's primary outcomes were incidence of quit attempts, incidence of self-reported 7-day cigarette abstinence, and the number and percent of participants in the intervention group who reported satisfaction with each intervention component. Secondary outcomes included intervention engagement and qualitative intervention feedback.

#### 2.2.5. Statistical Analysis

Data were analyzed in 2021 and 2022 using SPSS version 25. Descriptive statistics (e.g., means, standard deviations, and frequencies) were used to summarize participant characteristics and primary tobacco use outcomes. Logistic regressions were used to compare groups on the primary smoking outcomes using a penalized intent-to-treat (ITT) approach that classified survey nonrespondents as smokers and having not made a quit attempt. A two-sided *p* value < 0.05 was considered statistically significant. Descriptive statistics were used to summarize intervention engagement and satisfaction data. Illustrative quotes were selected from open-ended responses about participants' experiences with intervention components and with being encouraged to select a Monday quit day. Lastly, we conducted post hoc descriptive statistics characterizing 7-day abstinence rates and quit attempts among participants in the intervention group who did or did not use each intervention component.

## 3. Results

### 3.1. Recruitment and Retention

**Figure 1:**
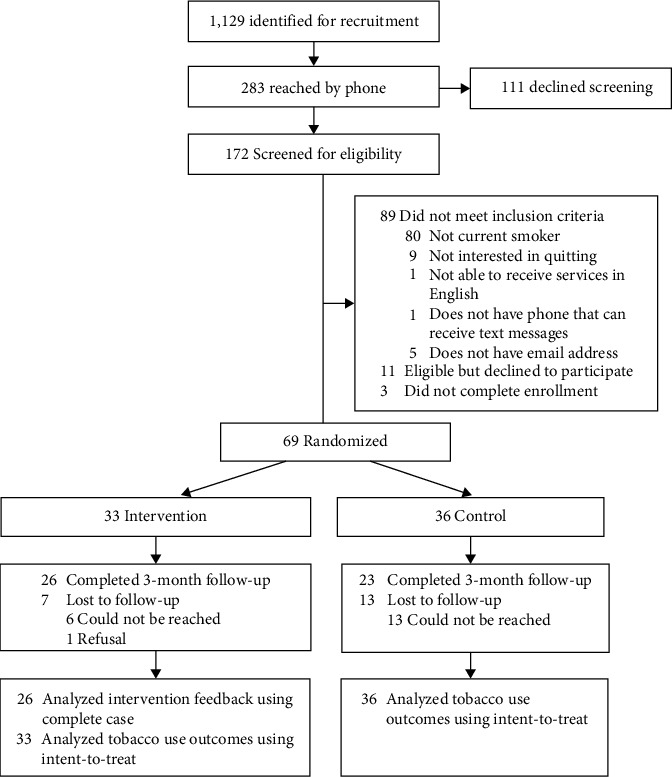
Flowchart of participant enrollment and follow-up. Notes: reasons for ineligibility do not add to 80, because participants may have indicated multiple reasons for ineligibility.

Figure 1 shows participant enrollment and retention. A sample of 100 was sought to provide 80% power (at *α* = 0.05) to statistically detect a 25% increase in predicted quit attempts in the intervention group versus control (75% vs. 50%). COVID-19-related staffing shortages and limits to patient contact resulted in a smaller sample size than planned. From February to September 2021, we screened 172 people for participation, 69 of whom met eligibility criteria, enrolled, and were randomized to intervention (*n* = 33) or control (*n* = 36).  Table 1 summarizes participant baseline characteristics. Participants were on average 54 (SD = 10.3) years old and were mostly female (57%), White race (63%), and non-Hispanic/Latinx ethnicity (75%). Participants smoked an average of 12.5 (SD = 9.2) cigarettes per day, and 93% were smoking every day. On scales of 0-10, participants scored an average of 7.7 (SD = 2.2) on motivation to quit and 5.6 (SD = 2.9) on confidence to quit. The most common mental health diagnoses in the sample were depression (34%), anxiety (27%), bipolar disorder (14%), and alcohol or substance abuse (3%). In total, 49 participants completed the 3-month follow-up assessments between May and December 2021 (71% response rate).

### 3.2. Smoking Outcomes

As shown in Table 2, intervention participants were more likely to have made a quit attempt at 3-month follow-up than control participants (64% vs. 39%, respectively; OR =2.75, 95% CI 1.03-7.30, and *p* = 0.04). Reported 7-day abstinence in intervention and control participants at 3 months was 12% and 6% (OR =2.35, 95% CI 0.40-13.74, and *p* = 0.35).

### 3.3. Intervention Engagement and Satisfaction

As shown in Table 3, 26 (79%) participants in the intervention group enrolled in the *QSQM* email newsletter, 24 (73%) began telephone coaching, 19 (58%) enrolled in the SmokefreeTXT program, and 15 (46%) used NRT. This compares to four (11%) control group participants who spoke with the NYS Quitline and six (17%) who used NRT by 3 months.

Table 4 displays quantitative and qualitative intervention feedback from the intervention group participants who responded to the 3-month survey. Of the 19 participants who recalled speaking with a counselor, 17 (89%) found the counseling to be very or somewhat helpful. Of the 13 participants who recalled receiving *QSQM* emails, nine (69%) found the emails to be very or somewhat helpful, nine (69%) felt that the number of emails they received was “about right” (compared to other response options of too many (*n* = 2) or not enough (*n* = 2)), and 100% (*n* = 13) were still receiving the emails at the time of the survey. Of the 14 participants who recalled enrolling in SmokefreeTXT, nine (64%) found the text messages to be very or somewhat helpful, 10 (67%) felt that the number of texts they received were “about right,” and eight (57%) had completed the texting program (seven opted-out of the program early).

When asked to provide open-ended feedback about what was helpful about the intervention components, positive themes emerged related to intervention *content*, while negative themes emerged related to the intervention *delivery* (Table 4). Participants viewed the telephone coach as a source of social and emotional support, accountability, and quitting tips and resources. Participants also found that the counselor helped them identify their reasons for smoking and reinforced that “every [quit attempt] was a new start.” Negative experiences with the coaching were related to the scheduling challenges. When asked to provide feedback about SmokefreeTXT, positive themes emerged related to the program's ability to provide encouragement, motivation, and reminders not to smoke. However, some participants felt that SmokefreeTXT was inflexible. For example, if a participant reported a relapse or having a slip, it was not easy to program a new Monday quit date. Two participants also shared that they felt that the frequent texts became smoking triggers. Regarding the *QSQM* emails, participants viewed the emails as a source of motivation and empowerment, as well as a positive reminder to start fresh each week. In contrast, some participants felt that they received too many emails or the emails were not effective communication.

**Figure 2:**
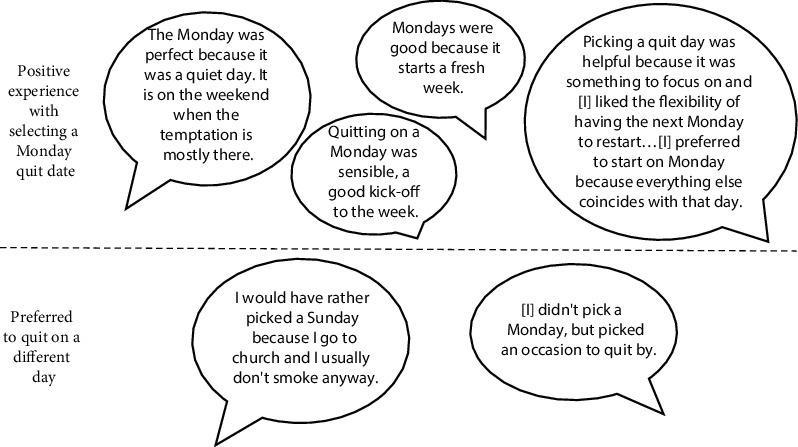
Example quotes from open-ended questions about intervention group participants' experiences with picking a Monday as their quit day (*n* = 12 survey respondents who had selected a quit date).

Lastly, 15 (45%) intervention participants selected a quit date during their time in coaching, 13 (87%) of whom selected a Monday quit date. Qualitative feedback on the follow-up survey about participants' experiences with being encouraged to pick a Monday quit day is shown in Figure 2. Of the 12 intervention group participants who reported setting a quit date on the follow-up survey, 10 reported positive experiences with selecting a Monday quit date, while two participants preferred to pick a different day.

### 3.4. Exploratory: Relationships between Intervention Engagement and Tobacco Outcomes

Our exploratory analyses examining self-reported abstinence and quit attempts among intervention group participants by whether they used or did not use each intervention component are shown in Table 5. Quit rates among people who did or did not use an intervention component were as follows: *QSQM* email (15% vs. 0%, respectively), telephone coaching (13% vs. 11%, respectively), SmokefreeTXT (11% vs. 14%, respectively), and NRT (7% vs. 17%, respectively). Quit attempts among people who did or did not use an intervention component were as follows: *QSQM* email (73% vs. 29%, respectively), telephone coaching (71% vs. 44%, respectively), SmokefreeTXT (74% vs. 50%, respectively), and NRT (80% vs. 50%, respectively).

## 4. Discussion

This study demonstrated the feasibility and acceptability of integrating the *QSQM* model into telephone coaching for people with MHCs. Our study further found that most people with MHCs enrolled in the *QSQM* email newsletter and had positive experiences with picking a Monday quit day. Qualitative feedback provided proof-of-concept that the *QSQM* newsletter and the Monday-anchored telephone coaching helped participants feel that quitting is a process with the opportunity to start fresh each week. Significantly more people in the intervention group made a quit attempt than people in the control group. Although the study was not powered to detect significant group differences in abstinence rates, the study's estimated intervention effect size on self-reported abstinence compared to referral to the Quitline was promising in relation to other smoking cessation interventions for people with MHCs [2, 14, 20–22]. The current study design and its small sample size precludes drawing conclusions about the working mechanisms of the intervention. Our exploratory analyses of quit rates and quit attempts stratified by participants' use of each intervention component (Table 5) suggest that enrollment in the weekly *QSQM* email newsletter may have been a significant intervention mechanism. However, it is also plausible that increasing access to behavioral cessation support, rather than the Monday-anchored quitting process specifically, improved tobacco outcomes. To isolate the impact of the *QSQM* approach on tobacco cessation, a future efficacy study should compare Monday-anchored behavioral support versus non-Monday-anchored behavioral support.

Although participants in the current trial found the *QSQM* model to be acceptable and encouraging, the study revealed intervention challenges that should be addressed before an efficacy study is attempted. The current trial had relatively low levels of intervention engagement. A recent systematic review by Perski et al. suggests that behavioral intervention engagement is primarily influenced by the intervention *context* (populations and settings) and elements of the *intervention itself* (content and delivery) [19]. With respect to populations and settings, low levels of engagement in mobile interventions and high rates of attrition are common in people with MHCs [17, 23, 24], potentially due to mental health symptoms (e.g., low mood or motivation and difficulty concentrating) or the avoidance of emotional reactions to being reminded of one's health problems [25]. This latter barrier was revealed in the current study when some participants shared that the frequent text messages and emails were reminders to smoke.

With respect to the intervention components themselves, qualitative feedback suggested that participant dissatisfaction with intervention delivery, rather than content, may have been a primary driver of low engagement. Participants wanted more contact with the telephone coach and found it difficult to attend the telephone sessions. Additionally, participants found SmokefreeTXT to be impersonal and inflexible, and almost half unsubscribed to the text messages early (consistent with previous evaluations of SmokefreeTXT [21, 26]). Regarding the *QSQM* email newsletter, the intervention counselor enrolled over 75% of participants in the *QSQM* newsletter, but only 54% of participants who responded to the follow-up survey recalled receiving the emails. This suggests that participants may not routinely check their email accounts, the emails went to a spam folder, or the emails simply went unnoticed. Formative work with people who have MHCs should be conducted to adapt or develop new *QSQM* delivery approaches that address the challenges identified in the current trial, while retaining and enhancing the intervention content that participants enjoyed (i.e., selecting a Monday quit day, framing quitting as a process and each week is an opportunity to start fresh, interpersonal support, empathy, accountability, and normalizing relapse). Future research may leverage mobile health (mhealth) delivery tools [27–29], including text-messaging systems and Smartphone apps, to deliver long-term personalized *QSQM*-related content and Monday-oriented coaching to people with MHCs. mHealth tools can overcome schedule-related barriers to counseling engagement by supporting both synchronous and asynchronous virtual coaching, including facilitated group discussions between people trying to quit. Recent reviews of smoking cessation mobile apps have identified several mhealth features that are associated with app popularity and use, including individual tailoring, the use of audio/visuals to deliver content, quit plan tracking, and proactive alerts [30, 31]. We recommend that investigators work closely with people who have MHCs to identify the most engaging, accessible, and efficacious mhealth platforms and features for delivering the *QSQM* model. Given that mhealth interventions have also been shown to be efficacious at reducing mental health symptoms [32–34], future research should examine whether integrating mental health-related content into *QSQM* content improves intervention engagement and outcomes among people with MHCs.

### 4.1. Limitations

Self-reported abstinence was not biochemically verified. The minimal control group does not allow us to determine whether anchoring the cessation process around Mondays was a significant intervention mechanism. The sample was predominantly female with a diagnosis of depression and/or anxiety and a high level of education, which may limit generalizability to other populations.

## 5. Conclusions

The study showed that the *QSQM* model was acceptable and potentially efficacious for people with MHCs, but there were barriers in the delivery of the approach using existing tools. Future research should work with people who have MHCs to adapt or develop new *QSQM* delivery tools that can be tested in a fully powered efficacy study.

## Figures and Tables

**Table 1:** Baseline characteristics of participants.

Variable	Total (*N* = 69)	Intervention (*n* = 33)	Control (*n* = 36)
Female, *n* (%)	39 (56.5%)	20 (60.6%)	19 (52.8%)
Age, mean (SD)	54.0 (10.3)	53.3 (10.5)	54.7 (10.2)
Race, *n* (%)			
White	43 (62.3%)	18 (54.5%)	25 (69.4%)
Black or African American	20 (29.0%)	11 (33.3%)	9 (25.0%)
Other	7 (10.1%)	5 (15.2%)	2 (5.6%)
Hispanic/Latinx ethnicity, *n* (%)	17 (24.6%)	6 (18.2%)	11 (30.6%)
Highest level of education, *n* (%)			
High school or less	15 (21.7%)	6 (18.1%)	9 (25.0%)
Some college or associate's degree	21 (30.4%)	13 (39.4%)	8 (22.2%)
4-year college or higher	33 (47.8%)	14 (42.4%)	19 (52.7%)
Marital status, *n* (%)			
Married or living with partner	26 (37.7%)	10 (30.3%)	16 (44.4%)
Divorced, separated, or widowed	13 (18.8%)	6 (18.2%)	7 (19.4%)
Never married	30 (43.5%)	17 (51.5%)	13 (36.1%)
Smoke every day, *n* (%)	64 (92.8%)	31 (93.9%)	33 (91.7%)
Cigarettes per day, mean (SD)	12.5 (9.2)	12.6 (7.8)	12.3 (10.5)
Quitting motivation (0-10), mean (SD)	7.7 (2.2)	8.0 (1.6)	7.4 (2.6)
Quitting confidence (0-10), mean (SD)	5.6 (2.9)	5.3 (2.6)	5.9 (3.2)
Time to first cigarette of the day, *n* (%)			
Within 5 minutes	25 (36.2%)	14 (42.4%)	11 (30.6%)
6-30 minutes	18 (26.1%)	7 (21.2%)	11 (30.6%)
31-60 minutes	13 (18.8%)	8 (24.2%)	5 (13.9%)
>60 minutes	13 (18.8%)	4 (12.1%)	9 (25.0%)

**Table 2:** Primary smoking outcomes at 3 months by group.

	Intervention (*n* = 33) *n* (%)	Control (*n* = 36) *n* (%)	OR (95% CI)
7-day cessation	4 (12.1%)	2 (5.6%)	2.35 (0.40-13.74); *p* = 0.345
Quit attempt	21 (63.6%)	14 (38.9%)	2.75 (1.03-7.30); *p* = 0.042

Notes: quit rates and quit attempt rates were calculated using a penalized intent-to-treat approach. Nonrespondents to the 3-month survey were classified as smokers and having not made a quit attempt. OR: odds ratio; CI: confidence interval.

**Table 3:** Participant use of smoking cessation treatment by group.

Variable	Intervention (*n* = 33) *n* (%)	Control (*n* = 36) *n* (%)
Enrolled in *QSQM* emails	26 (78.8%)	n/a
Spoke with study counselor	24 (72.7%)	n/a
Enrolled in SmokefreeTXT	19 (57.6%)	n/a
Used nicotine replacement therapy	15 (45.5%)	6 (16.7%)
Spoke with New York state Quitline	n/a	4 (11.1%)

Notes: *QSQM*: Quit and Stay Quit Monday.

**Table 4:** Intervention satisfaction and engagement among intervention group participants who responded to the follow-up survey.

Variable	*n* (%)	Positive themes regarding intervention *content* (example quotes)	Negative themes regarding intervention *delivery* (example quotes)
*Recalled speaking with a counselor*	**19 (79.2%)**	“The encouragement when I messed up. She encouraged me that it's not the end of world and just start over.” “Empathy and sensitivity. She had a great range of tools from behavioral changes to keeping records.” “I think the fact that the person was going to call and I think that the conversation about cigarettes make you think differently. To have a conversation makes me want to be accountable.”	“I needed two more sessions. The sessions needed to be longer. After two days past Monday, I picked up another cigarette. When I put Monday as my set date, I did not get a chance to come back to her. I needed two more weeks of counseling and support.”
Helpfulness of counseling			
Very	6 (31.6%)		
Somewhat	11 (57.9)		
Not at all	2 (10.5%)		

*Recalled receiving QSQM emails*	**13 (54.2%)**	“It lets you refresh yourself each time. With relapse and feeling badly, [the emails] were like ‘let us get back on the horse and do it again.'” “Reminded me that it is not always a one-time thing as far as quitting. You may have to take a process.” “It was reinforcing on days when I was feeling low and want to give up. I would read the emails and it would bring me back.”	“I would read it and skim through it, but it wasn't effective communication for me.” “Too many emails and constant reminder to smoke.”
Helpfulness of emails			
Very	4 (30.8%)		
Somewhat	5 (38.5%)		
Not at all	4 (30.8%)		
Frequency of emails			
About right	9 (69.2%)		
Too many	2 (15.4%)		
Too few	2 (15.4%)		
Long-term enrollment in emails			
Still receiving emails	13 (100.0%)		
Unsubscribed from emails early	0 (0.0%)		

*Recalled receiving text messages*	**14 (58.3%)**	“The messages were reassuring and supportive and provided a good point of view.” “It was a good alternative and helped me remain positive. I never had this option before and it just gives you something positive like how to channel something, simple things but it really helps reinforce it.” “It helps you not judge yourself and take things one day at a time. It was helpful to remind me to keep trying to quit.”	“The texts would remind me to start smoking. If you are in the moment of not smoking or thinking about it, it reminded me to smoke.” “Just that they are text messages—information was great but it would've been better if it was a person.” “Didn't work with a change of quit date.”
Helpfulness of texts			
Very	6 (42.9%)		
Somewhat	3 (21.4%)		
Not at all	5 (35.7%)		
Frequency of texts			
About right	10 (66.7%)		
Too many	2 (13.3%)		
Too few	3 (20.0%)		
Completion of texting program			
Completed texting program	8 (57.1%)		
Unsubscribed from texts early	6 (42.9%)		

**Table 5:** Self-reported 7-day abstinence and quit attempts at 3 months among participants in the intervention group who did and did not use a specific intervention component.

Intervention component	Participants who used the intervention component	Participants who did not use the intervention component
*7-day cessation*	**n** **(%) who quit**	**n** **(%) who quit**
*QSQM* email	4/26 (15.4%)	0/7 (0.0%)
Cessation counseling	3/24 (12.5%)	1/9 (11.1%)
SmokefreeTXT	2/19 (10.5%)	2/14 (14.3%)
Nicotine replacement therapy	1/15 (6.7%)	3/18 (16.7%)

*Quit attempt*	**n** **(%) who tried to quit**	**n** **(%) who tried to quit**
*QSQM* email	19/26 (73.1%)	2/7 (28.6%)
Cessation counseling	17/24 (70.8%)	4/9 (44.4%)
SmokefreeTXT	14/19 (73.7%)	7/14 (50.0%)
Nicotine replacement therapy	12/15 (80.0%)	9/18 (50.0%)

Notes: *QSQM*: Quit and Stay Quit Monday. Quit rates and quit attempt rates were calculated using a penalized intent-to-treat approach. Nonrespondents to the 3-month survey were classified as smokers and having not made a quit attempt. Independent *t*-tests showed that there were no significant differences between people who did or did not use each intervention component in these baseline measures (*p* > 0.05).

## Data Availability

De-identified data.
